# Mitochondrial Dysfunction in Diabetes: Shedding Light on a Widespread Oversight

**DOI:** 10.3390/pathophysiology32010009

**Published:** 2025-02-13

**Authors:** Franklyn Nonso Iheagwam, Amarachi Joy Joseph, Eniola Deborah Adedoyin, Olawumi Toyin Iheagwam, Samuel Akpoyowvare Ejoh

**Affiliations:** 1 Department of Biochemistry and Molecular Genetics, University of Colorado, Anschutz Medical Campus, Aurora, CO 80045, USA; 2 Department of Biochemistry, College of Science and Technology, Covenant University, Ota 112104, Nigeria; amarachi.joseph@stu.cu.edu (A.J.J.); eniola.adedoyinpgs@stu.cu.edu.ng (E.D.A.); 3 Genie Associates, Denver, CO 80238, USA; wuminaira@gmail.com; 4 Department of Biological Sciences, College of Science and Technology, Covenant University, Ota 112104, Nigeria; samuel.ejoh@covenantuniversity.edu.ng

**Keywords:** mitochondrial dysfunction, diabetes mellitus, insulin resistance, hyperglycemia, mitochondrial biogenesis, mitochondrial dynamics

## Abstract

Diabetes mellitus represents a complicated metabolic condition marked by ongoing hyperglycemia arising from impaired insulin secretion, inadequate insulin action, or a combination of both. Mitochondrial dysfunction has emerged as a significant contributor to the aetiology of diabetes, affecting various metabolic processes critical for glucose homeostasis. This review aims to elucidate the complex link between mitochondrial dysfunction and diabetes, covering the spectrum of diabetes types, the role of mitochondria in insulin resistance, highlighting pathophysiological mechanisms, mitochondrial DNA damage, and altered mitochondrial biogenesis and dynamics. Additionally, it discusses the clinical implications and complications of mitochondrial dysfunction in diabetes and its complications, diagnostic approaches for assessing mitochondrial function in diabetics, therapeutic strategies, future directions, and research opportunities.

## 1. Introduction

Diabetes mellitus (DM) is a prevalent metabolic disorder that poses significant health challenges worldwide and is characterised by chronic hyperglycaemia resulting from defects in insulin secretion, action, or both [1]. DM manifests in various forms, including type 1, type 2, gestational, prediabetes, and monogenic diabetes. Type 1 diabetes mellitus (T1DM) results from autoimmune destruction of pancreatic β-cells, resulting in absolute insulin deficiency, necessitating exogenous insulin therapy for glycaemic control. Genetic predisposition, environmental factors, and immune-mediated mechanisms contribute to the pathogenesis of T1DM, with polyuria, polydipsia, weight loss, and hyperglycaemia as clinical manifestations [2]. Type 2 diabetes mellitus (T2DM) occurs as a result of insulin resistance (IR) coupled with relative insulin deficiency and poses a threat to public health as it raises morbidity and mortality and causes poor quality of life. In addition to IR and impaired glucose homeostasis, genetic and environmental variables are also implicated in the aetiology of T2DM and impaired glucose uptake and metabolism in peripheral tissues and β-cell dysfunction [3,4]. Gestational diabetes mellitus (GDM) occurs during pregnancy due to inadequate insulin secretion or resistance. Hormonal changes, IR, and genetic factors contribute to the pathophysiology of GDM [5]. Prediabetes represents a crucial, reversible stage marked by elevated fasting plasma glucose (IFG) and/or impaired glucose tolerance (IGT) that can precede the onset of T2DM. Individuals with prediabetes frequently remain unaware of their elevated blood glucose levels because there are typically no noticeable symptoms until a diabetes diagnosis is made [6]. Mutations in a single gene that impact insulin action or β-cell function cause monogenic diabetes, not limited to maturity-onset diabetes of the young (MODY), neonatal DM (NDM), and mitochondrial DM [7].

Mitochondria, commonly known as the “powerhouses” of cells, perform essential roles in apoptosis, signalling, oxidation processes, and cellular energy consumption and balance. Mitochondria are vital for the proper functioning and survival of peripheral tissues, especially pancreatic β-cells [8], neurons [9], adipocytes, myocytes, and hepatocytes [10]. They play diverse roles in these cells, including managing metabolism, dynamics, proton leak, mitochondrial bioenergetics, calcium (Ca^2+^) regulation, structural integrity, and turnover/mitophagy [8]. This organelle is a major producer and receptor of reactive oxygen species (ROS), which in turn impairs its function, induces excessive ROS generation, and decreases mitochondrial electron transport chain (mETC) activity and ATP production [11]. Furthermore, mitochondrial DNA (mtDNA), which encodes for respiratory chain complexes, is prone to ROS-induced damage and mutations. This oxidative damage compromises mETC function and worsens energy failure, in addition to oxidative stress and dysfunctional mitochondria, which have been implicated in the pathogenesis of various diseases, including DM [12].

An imbalance in energy homeostasis is a key characteristic of individuals with DM, and substantial evidence has linked the development of DM to mitochondrial dysfunction [9]. Mitochondrial dysfunction (MD) affects oxidative phosphorylation, ROS production, mtDNA integrity, biogenesis, and dynamics. These abnormalities contribute to IR, β-cell dysfunction, and diabetic complications [13]. Therefore, addressing MD and DM crosstalk is crucial for understanding the biochemistry involved in maintaining cellular energy balance and improving DM symptoms [10]. This review aims to elucidate the complex link between mitochondrial dysfunction and diabetes, covering the spectrum of diabetes types, the role of mitochondria in insulin resistance, highlighting pathophysiological mechanisms, mitochondrial DNA damage, and altered mitochondrial biogenesis and dynamics. Additionally, it discusses the clinical implications and complications of mitochondrial dysfunction in diabetes and its complications, diagnostic approaches for assessing mitochondrial function in diabetics, therapeutic strategies, future directions, and research opportunities. Utilising search strategies, databases like PubMed, Scopus, and Google Scholar were queried using terms such as ‘mitochondria,’ ‘dysfunction’, ‘diabetes’, ‘diabetes mellitus’, ‘mitochondrial damage’, and ‘hyperglycaemia’ to locate pertinent literature published in the last decade.

## 2. Mitochondrial Dysfunction in Diabetes

**Figure 1:**
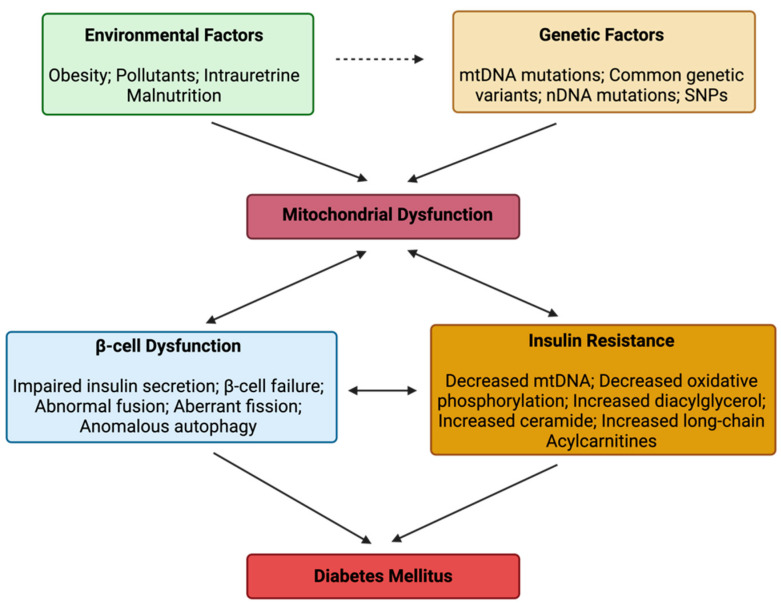
The relationship between mitochondrial dysfunction and diabetes mellitus.

Mitochondrial dysfunction plays a significant role in the pathogenesis of DM, contributing to IR, impaired glucose metabolism, and oxidative stress. It may arise due to genetic or environmental factors not limited to mutations, obesity and environmental pollutants, which propagate β-cell dysfunction and IR to drive DM onset and the downstream effect (Figure 1).

### 2.1. Mitochondrial Dysfunction-Induced Insulin Resistance and Impact on Glucose Metabolism

**Figure 2:**
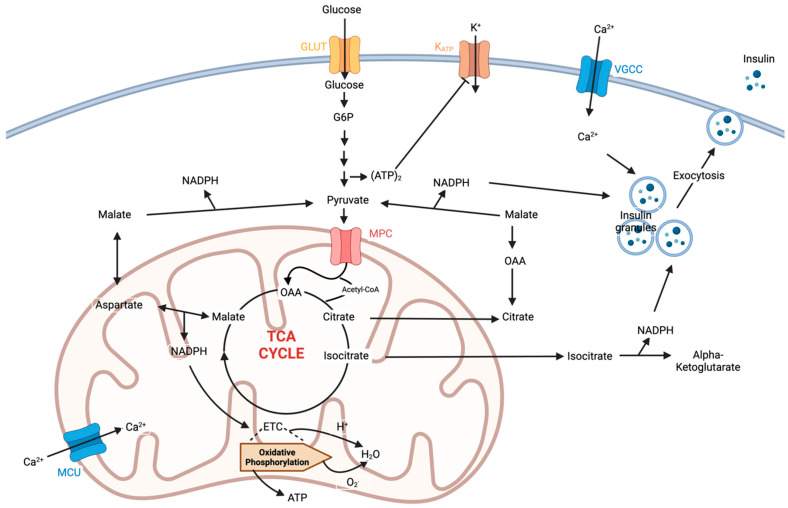
Mitochondrial pathways impacting insulin secretion and possible action. Abbreviations: TCA, tricarboxylic acid; OAA, oxaloacetate, ETC, electron transport chain; MCU, mitochondrial calcium uniporter; VGCC, voltage-gated calcium channels; GLUT, glucose transporters; MPC, mitochondrial pyruvate carrier.

Mitochondria are dynamic organelles involved in cellular energy production, the regulation of apoptosis, and various metabolic pathways, especially in insulin-sensitive tissues such as the skeletal muscle, the liver, and the adipose tissue. IR dysregulates glucose uptake and mitochondrial function and vice-versa, where oxidative phosphorylation (OXPHOS) and ATP production are the mechanisms through which MD contributes to IR, as shown in Figure 2 [14]. Reduced ATP synthesis impairs insulin signalling and phosphatidylinositol-3-kinase/Akt pathways, leading to decreased glucose uptake and glycogen synthesis, dysregulated fatty acid metabolism, and the accumulation of lipid intermediates, which further exacerbate IR by interfering with insulin signalling cascades and glucose metabolic pathways, further exacerbating hyperglycaemia [1,4]. MD leads to the accumulation of ROS, which activates stress kinases and inflammatory pathways, contributing to IR. Impaired mitochondrial biogenesis and dynamics also play a role in IR, as dysfunctional mitochondria fail to adapt to changing metabolic demands, leading to cellular dysfunction and IR [15].

### 2.2. Contribution of Reactive Oxygen Species (ROS)

Reactive oxygen species are byproducts of mitochondrial metabolism and perform two roles in DM: acting as signalling molecules at physiological levels and inducing oxidative stress and cellular damage at pathological levels. When excessive ROS are produced, this induces oxidative stress and cellular damage, leading to MD and impairment of insulin signalling pathways, ultimately causing IR [15,16]. ROS directly interfere with insulin signalling molecules, such as insulin receptor substrate (IRS) proteins, leading to impaired insulin signalling and glucose uptake [1]. Oxidative stress also induces mtDNA damage and mutations, further exacerbating MD and the generation of ROS, leading to a vicious cycle of oxidative stress and cellular damage [8]. Mitochondrial reactive oxygen species (ROS) are produced from several sources, each influencing mitochondrial oxidative stress with varying effects on muscle disorders and related diabetes conditions [17]. When NAD^+^ is scarce, α-Ketoglutarate Dehydrogenase (α-KGDH) produces ROS, especially in the skeletal muscle where NADH transhydrogenase (NNT) levels are low [18]. The 2-oxoacid dehydrogenase complexes are a group of mitochondrial enzymes that contain dihydrolipoamide dehydrogenase and generate superoxide and hydrogen peroxide while transferring reducing equivalents to FAD [19].

### 2.3. Genetic Factors Associated with Mitochondrial Dysfunction

**Figure 3:**
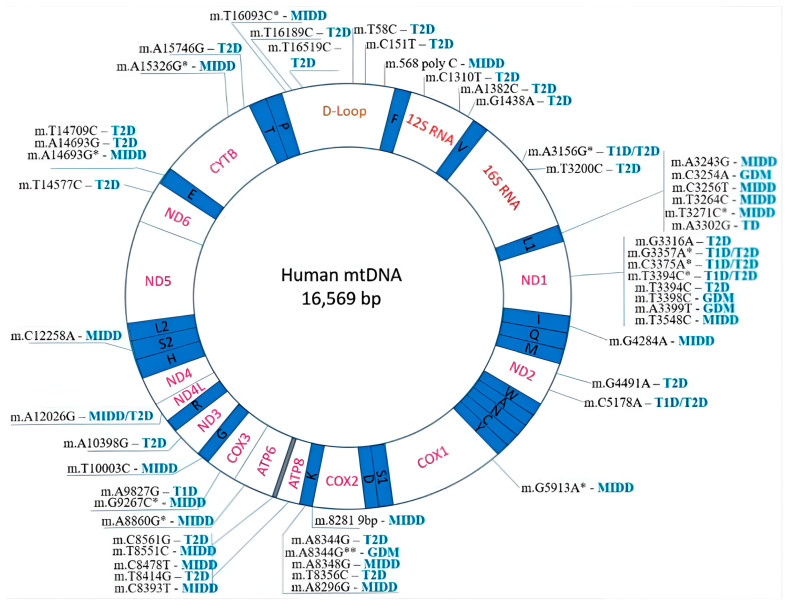
Map of mtDNA mutations associated with diabetes mellitus [23]. *: Newly discovered mutations; **: Also associated with a maternally inherited multisystem mitochondrial disorder known as myoclonic epilepsy with ragged red fibres (MERRF).

Genetic factors are significant in MD and DM pathogenesis, such as mutations in nuclear-encoded mitochondrial genes and mtDNA (Figure 3). The strong association between MD and IR is due to the unique structural attributes of mtDNA, which lead to a higher rate of mutations compared to nuclear DNA [20]. Genetic alterations in mtDNA, whether inherited or acquired, have been linked to the onset of IR, with their mutations often associated with diminished pancreatic β-cell function and impaired glucose-stimulated insulin secretion [21]. Several nuclear-encoded genes involved in mitochondrial biogenesis, oxidative phosphorylation, and mitochondrial dynamics have been implicated in DM susceptibility. Mutations in these genes disrupt mitochondrial function, leading to IR, impaired glucose metabolism, and DM development. Mutations in mtDNA, particularly in genes encoding mitochondrial tRNA and complex I subunits, have been linked to MD and DM [22]. These mutations impair mitochondrial protein synthesis and oxidative phosphorylation, leading to defective mitochondrial function and DM susceptibility. Recently, mtDNA mutation m.3243G and other mtDNA mutations have been associated with DM [23]. The homogenous A3302G mutation in the receptor arm of the tRNALeu^(UUR)^ gene disrupts a highly conserved base pairing (2T-71A), which impairs mitochondrial tRNA metabolism and contributes to IR [24]. Similarly, the down-regulation of mitochondrial genes such as MT-ND1, MT-ND2, MT-ND4, MT-ND4L, MT-ND5, MT-ND6, and MT-ATP6, which are crucial for OXPHOS and energy transduction, is linked to the pathogenesis of DM [25]. Disruptions in NAT2 and SLC16A11 genes cause reduced mitochondrial activity and MD, which is linked to decreased insulin sensitivity and ultimately leads to IR [26].

### 2.4. Gut Microbiome and Mitochondrial Dysfunction Interplay

The microbiota of the human gastrointestinal system sends signals to the mitochondria, and disturbances in this microbiota can alter mitochondrial metabolism, activate immune cells, and compromise the function of the epithelial barrier [27]. Additionally, the metabolites produced by these microbiota influence inflammasome activation and the production of inflammatory cytokines, which are significant contributors to DM. Such alterations in gut microbiota can exacerbate DM by inducing inflammation, disrupting glucose metabolism, and contributing to IR by impairing the gut mucosal barrier and increasing intestinal permeability. This permeability leads to the release of lipopolysaccharides (LPS) into the bloodstream, which activates the toll-like receptor 4 (TLR4) immune pathway and promotes chronic inflammation [28]. In response to microbial and inflammatory signals, phagocytes generate reactive oxygen species (ROS) within mitochondria. Additionally, increased intestinal permeability and the subsequent migration of immunogenic bacterial products intensify the inflammatory response. LPS/TLR interactions promote the production of cytokines, which activates tyrosine kinase and results in cyclooxygenase phosphorylation and diminished ATP production in mitochondria [29].

**Figure 4:**
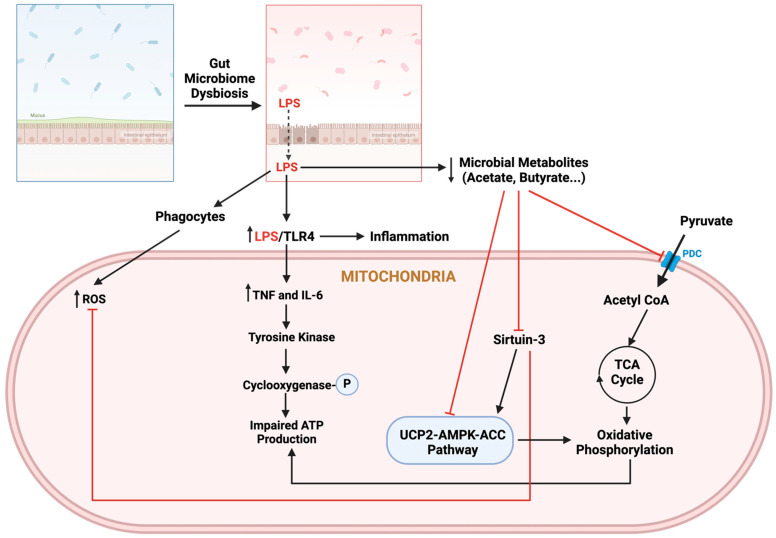
Microbiota–mitochondria interplay. Abbreviations: AMPK, AMP-activated protein kinase; COX, cyclooxygenase; LPS, lipopolysaccharide; ROS, reactive oxygen species; PDC, pyruvate dehydrogenase complex; ACC, acetyl-CoA carboxylase; UCP-2, uncoupling protein 2.

Dysbiosis affecting butyrate-producing bacteria is positively associated with glycemic disturbances by impacting the pathophysiology of DM [30]. This condition hampers butyrate’s ability to enhance mitochondrial function by decreasing the levels of SIRT-3 while truncating pyruvate dehydrogenase complex (PDC) activity. Consequently, reduced conversion of pyruvate to acetyl-CoA leads to impaired ATP production from the TCA cycle and OXPHOS [31]. Similarly, mitochondrial biogenesis is curtailed due to inhibited histone deacetylases, which diminishes energy expenditure. Dysbiosis also suppresses the production of acetate along with butyrate, causing MD and increased oxidative and nitrosative stress in pancreatic islets and β-cells. This suppression affects AMP-activated protein kinase (AMPK) activity, disrupting the regulation of mitochondrial OXPHOS [29]. The resulting reduction in the uncoupling protein 2 (UCP-2)-AMPK-acetyl-CoA carboxylase (ACC) pathway decreases the AMP ratio, leading to impaired glucose uptake and reduced mitochondrial OXPHOS efficiency (Figure 4).

## 3. Pathophysiology of Mitochondrial Dysfunction in Diabetes

**Figure 5:**
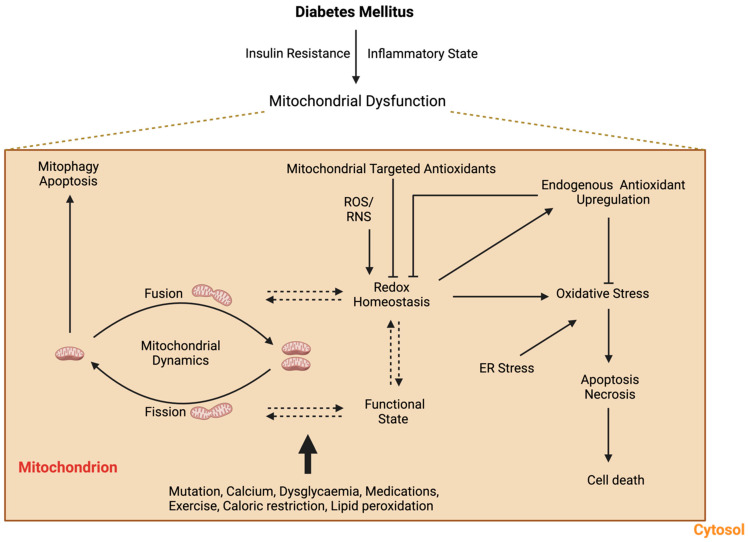
The interplay between diabetes mellitus, mitochondrial dysfunction and dynamics.

Mitochondrial dysfunction is a key feature in the pathophysiology of DM, contributing to the development and progression of the disease [12]. The interplay between mitochondrial dynamics, oxidative stress and mitochondrial function in a diabetic state is depicted in Figure 5.

### 3.1. Impaired Oxidative Phosphorylation

Oxidative phosphorylation is the primary mechanism by which mitochondria generate ATP, which is the currency of cellular energy. Reduced phosphorylation via oxidation reduces ATP production, contributing to dysfunction and metabolic abnormalities. Several factors contribute to impaired OXPHOS in DM, including dysregulated ETC activity, mitochondrial uncoupling, and substrate overload [14]. Dysfunctional ETC complexes, particularly complex I and III, disrupt electron flow and impair proton pumping across the inner membrane of mitochondria, reducing ATP synthesis [32]. Mitochondrial uncoupling proteins (UCPs), such as UCP1, UCP2, and UCP3, contribute to controlling the potential of the mitochondrial membrane and dissipating proton gradients [33]. In DM, upregulation of UCP expression uncouples oxidative phosphorylation from ATP synthesis, leading to increased energy dissipation and reduced ATP production [34]. Excessive nutrient intake, such as high-fat diets, results in substrate overload and MD. Elevated levels of fatty acids and glycaemia overwhelm mitochondrial capacity, leading to impaired substrate oxidation and ATP production [35].

### 3.2. Enhanced Reactive Oxygen Species (ROS) Production

Highly reactive chemicals known as reactive ROS are produced during mitochondrial metabolism, primarily through the ETC [32]. MD causes excessive production of superoxide anion (O_2_^•−^), hydrogen peroxide (H_2_O_2_), and hydroxyl radical (^•^OH), which are byproducts of incomplete electron transfer during oxidative phosphorylation that overwhelm cellular antioxidant defences, leading to oxidative stress and cellular damage [13]. Dysfunctional ETC complexes, particularly complex I and III, leak electrons to molecular oxygen-producing superoxide [32]. The pathophysiology of DM is influenced by lipid peroxidation, protein oxidation, and DNA damage, which damages cellular membranes, reduces enzyme activity, and causes mutations and cellular malfunction, respectively [36]. ROS-mediated oxidative stress activates stress kinases and inflammatory pathways, further exacerbating dysfunction and IR. The advancement of DM is facilitated by NF-κB and c-Jun N-terminal kinase pathways being activated, which enhance the production of pro-inflammatory cytokines and disrupt insulin signalling [16].

### 3.3. Mitochondrial DNA (mtDNA) Damage

Mitochondrial DNA (mtDNA) is vital to mitochondrial function and metabolism because it encodes critical elements that make up the ETC. mtDNA is particularly vulnerable to oxidative damage due to its proximity to the ETC and lack of protective histones. High ROS production levels lead to mtDNA damage, including base modifications, strand breaks, and deletions, compromising mitochondrial protein synthesis and OXPHOS function [37]. Accumulation of mtDNA mutations disrupts ETC activity, impairing ATP production and increasing ROS generation, creating a vicious cycle of oxidative stress and dysfunctional mitochondria [13]. Moreover, the relevance of mtDNA integrity in metabolic balance is further demonstrated by the correlation between mtDNA mutations, DM-associated complications, and mitochondrial disorders [38].

### 3.4. Altered Mitochondrial Biogenesis

The process by which cells grow the bulk and functionality of their mitochondria in response to environmental cues and metabolic demands is known as mitochondrial biogenesis. Alterations in mitochondrial biogenesis contribute to MD and metabolic abnormalities [39]. Transcriptional coactivators regulate mitochondrial biogenesis and function, such as peroxisome proliferator-activated receptor gamma coactivator-1 alpha (PGC-1α). Dysregulation of PGC-1α activity leads to impaired mitochondrial biogenesis and reduced mitochondrial gene expression in oxidative phosphorylation and energy metabolism [40]. Inflammatory cytokines and metabolic stressors, such as hyperglycaemia and dyslipidaemia, inhibit mitochondrial biogenesis and promote MD [36]. Activation of stress kinases and transcription factors, such as nuclear factor kappa B (NF-κB) and peroxisome proliferator-activated receptors (PPARs), disrupt mitochondrial biogenesis pathways, impairing mitochondrial function and contributing to metabolic abnormalities [41].

### 3.5. Dysfunctional Mitochondrial Dynamics

Mitochondrial dynamics refer to the processes of mitochondrial fusion, fission, and trafficking, which regulate mitochondrial morphology, distribution, and function [42]. Mitochondrial fusion enables the exchange of mitochondrial contents and promotes the integrity and functionality of the mitochondria. Impaired mitochondrial fusion leads to fragmented mitochondria and compromised mitochondrial function [43]. Conversely, excessive mitochondrial fission results in mitochondrial fragmentation and mitophagy, leading to the selective destruction of defective mitochondria. Dysregulated mitochondrial fission in DM exacerbates MD and oxidative stress, further impairing cellular metabolism and contributing to disease progression [44].

## 4. Clinical Implications and Complications of Mitochondrial Dysfunction in Diabetes

**Figure 6:**
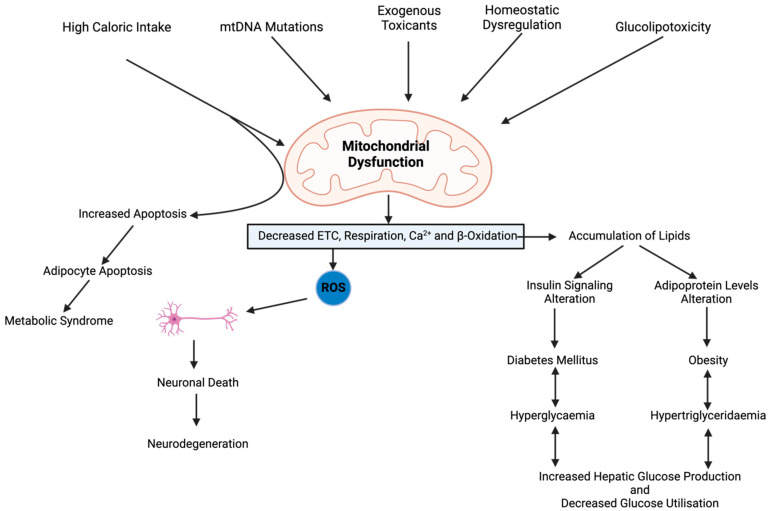
The association between mitochondrial dysfunction and clinical symptoms of diabetes and obesity. Abbreviations: ROS, reactive oxygen species; mtDNA, mitochondrial DNA; ETC, electron transport chain.

A spectrum of clinical implications and complications associated with DM arise from mitochondrial dysfunction, not limited to insulin intolerance and β-cell damage, which are common occurrences in addition to various systemic complications affecting the cardiovascular, renal, neurological, and ocular systems (Figure 6).

### 4.1. Insulin Resistance and β-Cell Dysfunction

The aetiology of IR involves a complex interplay between genetic, environmental, and lifestyle factors, including obesity, physical inactivity, dietary habits, and genetic predisposition. Adipose tissue-derived cytokines, such as tumour necrosis factor-alpha and interleukin-6 (IL-6), promote IR by interfering with insulin signalling pathways and promoting inflammation [45]. These occurrences drive β-cell dysfunction, characterised by impaired insulin secretion and reduced β-cell mass, further exacerbating hyperglycaemia and metabolic dysfunction. Chronic exposure to hyperglycaemia, lipotoxicity, and glucotoxicity impairs β-cell function and viability, leading to progressive deterioration of insulin secretion capacity [1,4]. MD significantly contributes to pancreatic β-cell failure, IR and DM when chronic hyperglycemia heightens these cells’ metabolic demands, causing excessive ROS production [46]. Persistent ROS generation leads to oxidative stress and apoptosis of β-cells, resulting in reduced β-cell mass, which, in genetically predisposed individuals, accelerates the transition from IR to DM [47].

### 4.2. Diabetic Cardiomyopathy

Diabetic cardiomyopathy is a distinct form of heart disease characterised by structural and functional abnormalities in the myocardium, independent of traditional cardiovascular risk factors. Diabetes-associated metabolic disturbances, including hyperglycaemia, IR, dyslipidaemia, and oxidative stress, contribute to the development of diabetic cardiomyopathy [48]. Hyperglycaemia promotes myocardial fibrosis, hypertrophy, and cell death, leading to diastolic and systolic dysfunction in diabetic cardiomyopathy. IR and dyslipidaemia impair myocardial energy metabolism and calcium handling, further exacerbating contractile dysfunction and heart failure [49]. Oxidative stress and inflammation also play pivotal roles in the pathogenesis of diabetic cardiomyopathy, promoting myocardial injury and remodelling. Increased production of ROS and pro-inflammatory cytokines, such as IL-1β and IL-6, contribute to oxidative damage, inflammation, and myocardial dysfunction [50]. In diabetic cardiomyopathy, MD increased fatty acid oxidation leads to mitochondrial uncoupling, characterised by elevated proton leak and impaired ATP regeneration [51]. This process is driven by fatty acid-induced mitochondrial uncoupling and increased activity of uncoupling proteins (UCPs), which are further activated by elevated mitochondrial ROS [51]. Additionally, reduced mitochondrial Ca^2+^ uptake and Ca^2+^ mishandling contribute to decreased ATP production and enhanced ROS generation [52]. Impaired interactions between inositol trisphosphate (IP3) receptors and voltage-dependent anion channels (VDACs) lead to reduced IP3-stimulated Ca^2+^ transfer to mitochondria. This misalignment in Ca^2+^ transfer contributes to lower mitochondrial energy production and compromised cell contraction [53].

### 4.3. Diabetic Nephropathy

A typical microvascular consequence of DM is diabetic nephropathy, which is characterised by a gradual loss of renal function and the emergence of albuminuria, ultimately leading to end-stage renal disease [54]. The pathogenesis of diabetic nephropathy involves multifactorial mechanisms, including hemodynamic alterations, metabolic disturbances, and inflammatory processes [55]. Chronic hyperglycaemia and systemic IR contribute to glomerular hyperfiltration, hypertension, and renal hypertrophy in diabetic nephropathy. Increased intraglomerular pressure and activation of the renin–angiotensin–aldosterone system promote glomerular injury and sclerosis, leading to a progressive decline in renal function [56]. Mitochondrial dysfunction significantly impacts DN through mtDNA damage due to its proximity to ROS and lack of histone protection, which leads to systemic inflammation and renal dysfunction. Additionally, mitochondrial dynamics become dysregulated with impaired mitophagy, exacerbating MD and DN [57]. In diabetic nephropathy, the production of ATP primarily occurs through OXPHOS in dysfunctional mitochondria, with fatty acids, lactate, and glutamine as key substrates for ATP production in the proximal convoluted tubules [58]. Increased delivery of these substrates leads to increased ROS and altered energy expenditure, contributing to proximal tubular cell damage, tubular atrophy, poor clinical outcomes, and DN progression [59]. Mitochondrial dysfunction worsens DN due to increased levels of mitogen-activated protein kinase 1 (MAPK1), which lowers phosphofurin acidic cluster sorting protein 2 (PACS-2) levels, a key element of the mitochondria-associated endoplasmic reticulum membrane (MAM) responsible for tethering mitochondria to the endoplasmic reticulum and causing mitochondrial fragmentation [60]. Metabolic factors, such as dyslipidaemia, advanced glycation end-products, and oxidative stress, contribute to renal inflammation, fibrosis, and tubulointerstitial damage in diabetic nephropathy [61]. Activation of pro-inflammatory pathways, including nuclear factor kappa B (NF-κB) and transforming growth factor-beta (TGF-β), exacerbates renal injury and impairs renal function [62].

### 4.4. Diabetic Neuropathy

Diabetic neuropathy (DN) encompasses a diverse group of peripheral nerve disorders associated with DM, characterised by sensory, motor, and autonomic dysfunction [63]. The pathogenesis of diabetic neuropathy involves multiple mechanisms, including metabolic, vascular, and inflammatory processes [64]. Downregulation of sirtuin-1 (SIRT1) disrupts nerve function and triggers neuropathic symptoms by impairing mitochondrial biogenesis through the suppression of PPARG coactivator 1 alpha and disrupting antioxidant signalling mediated by NFE2-like bZIP transcription factor 2 [65]. Activation of poly (ADP-ribose) polymerase 1 decreases mitophagy and is linked to mitochondrial damage in dorsal root ganglion neurons, resulting in mitochondrial oxidative damage and neuropathic pain [66]. Hypoxia-inducible factor 1 subunit alpha downregulation during DN onset modifies the PRKN signalling pathway and reduces PRKN protein levels, disrupting mitophagy, which leads to increased mitochondrial dysfunction and hyperalgesia [67]. Chronic hyperglycaemia and dyslipidaemia lead to metabolic disturbances and oxidative stress, contributing to nerve damage and dysfunction in diabetic neuropathy. Impaired glucose metabolism disrupts nerve fibre structure and function, leading to axonal degeneration, demyelination, and impaired nerve conduction [68]. Inhibition of neurite growth is also a consequence of impaired glucose metabolism and MD [65]. Vascular abnormalities, including microangiopathy and endothelial dysfunction, further exacerbate nerve ischemia and hypoxia in diabetic neuropathy. Reduced blood flow and impaired neurovascular coupling compromise nerve perfusion and oxygenation, contributing to nerve injury and neuropathic symptoms [69]. The effects of mitochondrial dysfunction in DM extend beyond the nervous system, as reduced AMPK activity and lower expression of mitochondrial complex proteins have been noted in kidney mesangial cells in diabetic nephropathy [70].

### 4.5. Diabetic Retinopathy

Diabetic retinopathy (DR) is a common microvascular complication of DM, characterised by progressive damage to the retinal microvasculature, leading to visual impairment and blindness. In DR, retinal MD involves a complex interplay of structural, functional, and genomic abnormalities, resulting in inhibited complex I and III activities, increased free radical production, and reduced SOD2 levels [71]. In diabetic retinas, NADPH oxidase 2 activation precedes mitochondrial damage, with persistent increases in cytosolic ROS contributing to mitochondrial injury; elevated ROS levels induce mitochondrial dysfunction by damaging mitochondrial components and intensifying oxidative stress [72]. Matrix Metalloproteinase 2 (MMP-2) and 9 (MMP-9) are upregulated, with MMP-9 activation occurring early in the damage process [73]. Homocysteine exacerbates this by increasing ROS production, which activates MMP-9 and reduces its inhibitor, Timp1, damaging mitochondria and inducing apoptosis of retinal microvascular cells [74]. It also reduces mitochondrial transcription factor A, further compromising mitochondrial function by exacerbating mtDNA damage and impairing mtDNA biogenesis [75]. Chronic hyperglycaemia and systemic metabolic disturbances contribute to retinal microvascular dysfunction and neurodegeneration in diabetic retinopathy. Prolonged exposure to hyperglycaemia promotes retinal vascular leakage, capillary non-perfusion, and the formation of microaneurysms in diabetic retinopathy. Increased oxidative stress and inflammation exacerbate retinal vascular injury and disrupt blood–retinal barrier integrity, leading to retinal oedema and neovascularisation [76]. Angiopoietin-2 (Ang-2) and vascular endothelial growth factor are two dysregulated angiogenic factors that induce aberrant neovascularisation and fibrosis in diabetic retinopathy. In more advanced stages of the illness, activating pro-fibrotic pathways, such as connective tissue growth factor and transforming growth factor- β, contributes to retinal fibrosis and vision loss [77].

## 5. Diagnostic Approaches and Biomarkers

Accurate DM diagnosis and monitoring of MD are essential for effective management and intervention strategies. The clinical assessment of DM is well elucidated; however, evaluating MD during its onset is often overlooked. MD can be diagnosed and identified using respiratory chain enzyme assays, mtDNA analysis, metabolomic and proteomic profiling, and imaging techniques [14].

### 5.1. Respiratory Chain Enzyme Assays

Respiratory chain enzyme assays are biochemical tests used to evaluate the activity of individual ETC complexes, providing insights into mitochondrial function and integrity [78]. Impaired respiratory chain enzyme activity shows MD and OXPHOS defects, contributing to metabolic abnormalities and DM progression [79]. Reduction in ETC complex activity, particularly complex I and complex III, is commonly observed in diabetic tissues, leading to decreased ATP production and increased ROS generation [80]. Respiratory chain enzyme assays can be performed using tissue biopsies or cultured cells from diabetic patients to assess mitochondrial function and metabolic status. These assays provide valuable information for diagnosing DM-induced mitochondrial disorders and vice-versa, monitoring disease progression, and evaluating therapeutic responses [81].

### 5.2. Mitochondrial DNA Analysis

Mitochondrial DNA analysis examines mitochondrial genome integrity, mutations, and copy number alterations, providing insights into MD and genetic susceptibility in DM [38]. mtDNA mutations and deletions are associated with DM and its complications [37]. mtDNA analysis can identify pathogenic mutations and polymorphisms that associate MD with T1DM and T2DM. Common mtDNA variants, such as the m.A3243G in the mitochondrial tRNA^Leu^ gene, are implicated in mitochondrial diseases and DM, highlighting the importance of genetic testing and risk assessment [23]. Quantitative analysis of mtDNA copy number, measured using real-time polymerase chain reaction, provides additional information on mitochondrial biogenesis and function. An altered mtDNA copy number is observed in diabetic tissues, reflecting MD and metabolic stress in reaction to hyperglycaemia and oxidative stress [38]. mtDNA analysis is a valuable diagnostic tool for identifying individuals at risk of developing DM and its complications, facilitating personalised management and intervention strategies based on genetic predisposition and metabolic status [23].

### 5.3. Metabolomic Profiling

Metabolomic profiling involves the comprehensive analysis of small-molecule metabolites in biological samples, such as blood, urine, and tissues, to characterise metabolic pathways and identify biomarkers associated with disease states [82]. Metabolomic profiling offers insights into systemic metabolic abnormalities and MD, enabling early detection and intervention. Metabolomic studies have identified distinct metabolic signatures connected to insulin intolerance, β-cell dysfunction, and diabetic complications, including alterations in amino acid metabolism, lipid metabolism, and oxidative stress pathways [83]. Metabolomic profiling enables the identification of novel therapeutic targets and intervention strategies for mitigating metabolic disturbances and improving clinical outcomes in DM [82]. Targeted metabolomics and flux analysis techniques provide mechanistic insights into mitochondrial metabolism and energy homeostasis, assisting in developing personalised treatment approaches tailored to individual metabolic profiles. Dysregulated metabolites, such as branched-chain amino acids, acylcarnitines, and ceramides, are implicated in IR and MD, thus potentially being biomarkers for DM diagnosis and prognosis [84].

### 5.4. Imaging Techniques

Imaging techniques, such as positron emission tomography (PET), magnetic resonance imaging (MRI), and near-infrared spectroscopy (NIRS), are utilised to visualise mitochondrial function and tissue metabolism in vivo, offering non-invasive diagnostic modalities for assessing MD in DM [85]. PET imaging with radio-labelled tracers, such as [18F]fluorodeoxyglucose (FDG) and [11C]acetate, enables the assessment of tissue glucose uptake and oxidative metabolism, providing insights into mitochondrial function and energy metabolism in diabetic tissues [86]. Altered FDG uptake and mitochondrial oxidative capacity are observed in insulin-resistant tissues, reflecting MD and metabolic dysregulation. MRI techniques, including magnetic resonance spectroscopy and diffusion-weighted imaging, allow for the quantification of tissue metabolites and water diffusion properties, providing information on mitochondrial function and tissue microstructure [87]. Changes in tissue metabolite concentrations and diffusion parameters are associated with MD, inflammation, and fibrosis in diabetic tissues, serving as imaging biomarkers for disease progression and therapeutic response. NIRS imaging measures tissue oxygenation and blood flow, offering insights into mitochondrial respiration and microvascular dysfunction. Altered NIRS parameters, such as tissue oxygen saturation (StO_2_) and oxygen extraction fraction (OEF), are indicative of impaired mitochondrial oxygen consumption and tissue perfusion in diabetic patients, highlighting the importance of assessing mitochondrial function in the context of tissue oxygenation and metabolism [88].

### 5.5. Biomarkers

Biological indicators play a crucial role in disease management, particularly through their application in diagnostics and screening. Various biomarkers are instrumental in detecting MD in diabetes, especially in cases that are either asymptomatic or not yet exhibiting overt symptoms [89]. These biomarkers offer valuable information regarding disease prognosis, such as the likelihood of disease progression or complications, and can also predict therapeutic outcomes by distinguishing between patients likely to benefit from specific treatments and those who might not [89]. Furthermore, pharmacodynamic biomarkers are useful for identifying patients who will respond biologically to medical interventions, while safety biomarkers help pinpoint those at heightened risk of adverse effects from treatments [90]. There is an urgent need to classify prognostic biomarkers for individuals with DM who are at risk of MD, enabling targeted prevention and management strategies. Given the variability in treatment responses among individuals, predictive and response biomarkers are essential for making informed clinical decisions regarding treatment options [91]. Additionally, identifying prognostic biomarkers for complications associated with MD in DM—such as organ failure, microbiota imbalance, and mortality—is critical for selecting medications that could mitigate these risks. Identifying safety biomarkers for these treatments is also vital, as some therapies come with severe side effects that could lead to significant harm or even fatal outcomes [92]. Table 1 presents a selection of biomarkers with valuable MD prognoses from the discussed diagnostic approaches.

## 6. Therapeutic Strategies

MD is a pivotal factor in the pathogenesis of DM, contributing to metabolic abnormalities and cellular dysfunction. Targeted therapeutic interventions aimed at preserving mitochondrial function and mitigating oxidative stress hold promise for managing DM and its complications [16].

### 6.1. Mitochondrial-Targeted Antioxidants

**Figure 7:**
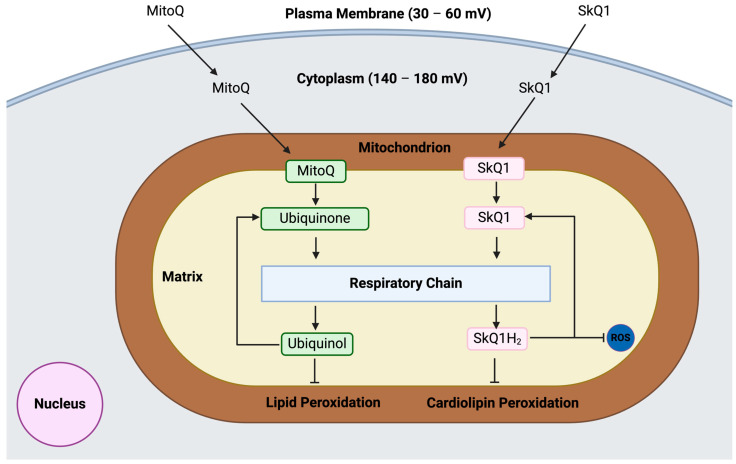
Antiperoxidation and radical scavenging mechanism of 10-(4,5-dimethoxy-2-methyl-3,6-dioxo-1,4-cyclohexadien-1-yl)decyl) triphenylphosphonium mesylate (MitoQ) and 10-(6′-plastoquinonyl) decyltriphenylphosphonium (SkQ1) in the mitochondria.

Antioxidant chemicals can suppress oxidation at many phases, including radical chain breaking (scavenging) and chelating metals as catalysts. Antioxidants that target mitochondria offer a potential treatment strategy for DM that reduces oxidative stress and maintains mitochondrial function [103]. These antioxidants accumulate within mitochondria, scavenging ROS and reducing oxidative damage to mitochondrial components [104]. MitoQ, a mitochondria-targeted derivative of coenzyme Q10, has demonstrated efficacy in preclinical and clinical studies for lowering oxidative stress and enhancing diabetic mitochondrial health [105]. Other mitochondrial-targeted antioxidants, such as SS-31 (elamipretide) and SkQ1, have shown promising results in preclinical studies for ameliorating MD and diabetic complications [106]. These compounds exhibit potent antioxidant properties and improve mitochondrial bioenergetics, suggesting their potential as adjunctive therapies for DM management (Figure 7). Resveratrol has been recognised as a Sirt1-activating compound (STAC) that enhances mitochondrial biogenesis and turnover and OXPHOS capacity [107]. Supplementing resveratrol with other antioxidants of natural sources, such as polyphenols, improves the respiratory activity of complexes I and II and enhances electron transport efficiency within the OXPHOS system. However, this supplementation does not influence mtDNA copy number, mitochondrial content, or the expression of respiratory chain proteins [108]. Mito-Tempo is a cell-permeable mimetic of superoxide dismutase (SOD) designed to specifically target mitochondria and neutralise free radicals through the conjugation of piperidine nitroxide with the triphenylphosphonium (TPP) group. This compound facilitates the conversion of superoxide anions (O_2_^•−^) into oxygen (O_2_) and hydrogen peroxide (H_2_O_2_), thereby mitigating oxidative stress in mitochondria associated with diabetes complications [109]. Notably, Mito-Tempo reduces oxidative stress, apoptosis, hypertension, and ROS production induced by hyperglycemia through modulation of the ERK1/2 signalling pathway and the GLP-1/CREB/adiponectin axis [110,111]. Additional mechanisms include the reduction in mitochondrial ROS in arterioles of diabetic individuals, which improves endothelial function, and the restoration of mitochondrial complex II activity impaired by IR [112]. Mito-PBN has also emerged as a promising antioxidant specifically targeting mitochondria, effectively scavenging free radicals produced within the mitochondria. When encapsulated in liposomes, Mito-PBN scavenges mitochondrial O_2_^•−^ and H_2_O_2_, leading to an improved NADH^+^ ratio, enhanced mitochondrial oxidative energy coupling, and increased ATP production, which in turn alleviates ROS-induced MD [113].

### 6.2. Metabolic Modulators

Metabolic modulators represent a diverse class of therapeutic agents that target cellular metabolism and mitochondrial function in DM. These agents exert favourable effects on insulin sensitivity, glucose homeostasis, and energy metabolism, offering potential therapeutic benefits for DM management [114]. Metformin, a first-line oral antidiabetic medication, exerts its therapeutic effects by inhibiting mitochondrial complex I activity, increasing insulin sensitivity and glucose uptake by activating adenosine monophosphate-activated protein kinase. Metformin modulates mitochondrial dynamics and biogenesis, contributing to its metabolic benefits in DM onset [115]. Additionally, sodium-glucose cotransporter 2 (SGLT2) inhibitors, such as dapagliflozin and empagliflozin, have emerged as promising therapeutic agents for DM management. These substances cause glucosuria and prevent renal glucose reabsorption, which results in calorie loss and metabolic adaption. SGLT2 inhibitors also benefit mitochondrial function and oxidative stress, contributing to their cardiorenal protective effects [116]. Imeglimin is a novel pharmacological agent aimed at reducing glucose levels and HbA1c, and it is currently undergoing evaluation in clinical trials, including several recently completed phase III studies with few side effects reported [117]. The primary mechanism of imeglimin’s action targets mitochondrial function. Research indicates that imeglimin administration reduces respiration linked to complex I substrates, while respiration associated with complex II substrates is enhanced [118]. Additionally, imeglimin elevates the protein content and enzymatic activity of complex III by inhibiting complex I, thus restoring complex III functionality and promoting complex II-linked respiration. This process may enhance fatty acid oxidation and decrease intrahepatic lipid accumulation, mitigating IR and improving insulin sensitivity [107]. Furthermore, imeglimin treatment has been shown to lower reactive oxygen species (ROS) production in complex II by inhibiting the formation of permeability transition pores, with no observed impact on overall mitochondrial respiration [119].

### 6.3. Gene Therapies

**Figure 8:**
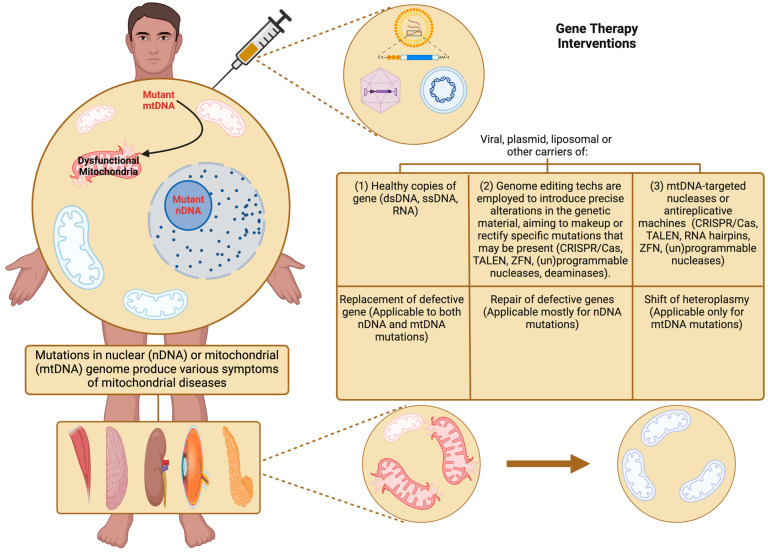
Gene therapy of mitochondrial diseases.

DM-associated mtDNA mutation treatments are currently insufficient, as they only alleviate symptoms without addressing the root cause or offering a cure [120]. Gene therapies hold significant promise for treating MD in DM as they aim to restore mitochondrial function, enhance cellular bioenergetics, and alleviate metabolic abnormalities through targeted manipulation of gene expression and mtDNA integrity [121]. Mitochondrial gene therapy, including allotopic expression and mitochondrial gene editing, offers potential strategies for correcting mtDNA mutations and restoring mitochondrial function, tackling the problem at its root [122,123]. Allotopic expression involves nuclear translocation of mitochondrial genes, bypassing mtDNA mutations and restoring protein synthesis within mitochondria. Mitochondrial gene editing technologies, such as CRISPR-Cas9, enable precise modification of mtDNA sequences, offering potential therapeutic benefits for inherited mitochondrial diseases and metabolic disorders [124]. CRISPR-based approaches hold promise for correcting mtDNA mutations associated with DM and restoring mitochondrial function in affected individuals [125]. These therapeutic approaches centre on introducing genetic material into the mitochondria to inhibit, modify, or enhance the function of malfunctioning genes (Figure 8). To facilitate the direct delivery of target genes into the mitochondria, vectors designed with high targeting specificity are loaded with genetic material [126]. These vectors are engineered to transport, shield, and guide the genetic content to the mitochondria, ensuring its effective release and promoting the expression of mitochondrial genes and their corresponding proteins [127]. Viral vectors, including retroviruses, adenoviruses, and lentiviruses, are extensively utilised in gene delivery due to their effectiveness in cell transfection [128]. Adeno-associated viruses (AAVs) are preferred in preclinical applications because of their substantial loading capacity, biocompatibility, low immunogenicity, and minimal immune response. Additionally, gene release research has frequently employed non-viral delivery systems that utilise cell-penetrating peptides, micelles, polymers, and lipids [129]. Moreover, ternary non-viral systems that combine polymers and/or peptides have demonstrated considerable efficacy in enhancing payload encapsulation, thus improving gene delivery and expression [130].

### 6.4. Lifestyle Interventions

Lifestyle interventions, including dietary modifications, physical activity, and weight management, are crucial in managing DM and its associated complications. These interventions target underlying metabolic abnormalities and promote mitochondrial health by modulating energy balance and substrate utilisation [15]. Low-carbohydrate, ketogenic, and intermittent fasting regimens have gained attention for their potential advantages in increasing mitochondrial function and metabolic health. These dietary approaches promote ketone body production, enhance mitochondrial biogenesis, and improve insulin sensitivity, offering therapeutic benefits for DM management [131]. Frequent exercise and physical activity also positively impact mitochondrial function, oxidative stress, and insulin sensitivity. Exercise promotes mitochondrial biogenesis, enhances mitochondrial quality control mechanisms, and enhances glucose absorption and utilisation, contributing to metabolic adaptation and disease management [132]. Exercise mimetics are compounds that replicate the physiological effects of physical exercise by engaging analogous signalling pathways, thereby enhancing mitochondrial content and mitigating oxidative stress within the mitochondria [107]. Substances such as 5-Aminoimidazole-4-carboxamide ribotide, GW501516, and various natural compounds present in certain foods, like epicatechin, have been identified as potential pharmacological aids for exercise via AMPK and PPAR activation [133]. These substances are particularly valuable for individuals unable to engage in physical activity due to health conditions.

## 7. Future Directions and Research Opportunities

The landscape of DM research is continuously evolving, with emerging technologies and approaches offering new insights into the pathophysiology of the disease and potential therapeutic targets. Advancements in omics technologies, encompassing genomes, transcriptomics, proteomics, and metabolomics, have transformed the molecular mechanisms underlying DM [134]. High-throughput sequencing techniques enable comprehensive analysis of genetic variants, gene expression profiles, protein abundance, and metabolite levels associated with DM and its complications [135]. Single-cell omics approaches provide unprecedented resolution and granularity, allowing researchers to dissect cellular heterogeneity and identify novel cell types, subpopulations, and signalling pathways involved in DM pathogenesis. Integrating multi-omics data sets offers a systems-level perspective of DM, unravelling complex networks of molecular interactions and regulatory circuits underlying disease progression [136].

In addition to omics technologies, advances in imaging modalities, such as MRI, PET, and optical imaging, enable non-invasive visualisation and quantification of metabolic, functional, and structural changes. Molecular imaging probes targeting specific biomarkers and metabolic pathways provide valuable insights into disease mechanisms and treatment responses in preclinical and clinical settings [85]. Despite significant progress in DM research, numerous unanswered questions and knowledge gaps persist, hindering the ability to develop effective prevention and treatment strategies. Key areas requiring further investigation include the role of epigenetics, environmental factors, and the microbiome in DM susceptibility and progression. Moreover, the interaction between environmental cues and genetic predisposition remains incompletely understood, highlighting the need for comprehensive multi-omics studies and large-scale epidemiological analyses to identify gene–environment interactions and modifiable risk factors for DM [137]. Furthermore, the heterogeneity of DM phenotypes and clinical outcomes poses a challenge for personalised medicine approaches, necessitating robust biomarkers and predictive models to stratify patients based on disease subtypes, prognosis, and treatment response [138].

## 8. Conclusions

This review highlights the intricate relationship between mitochondrial dysfunction and DM, emphasising mitochondria’s pivotal role in insulin resistance, glucose metabolism, and diabetic pathophysiology. Mitochondrial dysfunction leads to impaired oxidative phosphorylation, increased ROS production, mtDNA damage, and altered mitochondrial dynamics, exacerbating metabolic dysregulation and contributing to diabetic complications. Targeting mitochondrial dysfunction offers promising therapeutic avenues, including mitochondrial-targeted antioxidants, metabolic modulators, gene therapies, and lifestyle interventions. Diagnostic methods such as respiratory chain enzyme assays, mtDNA analysis, metabolomic profiling, and imaging enable early detection and personalised treatment strategies. Future research should address knowledge gaps through multi-omics studies to unravel the molecular mechanisms underlying mitochondrial dysfunction and DM across diverse populations.

## Figures and Tables

**Table 1:** Selected biomarkers for mitochondrial dysfunction prognosis.

Diagnostic Approach	Biomarker	Ref.
Respiratory Chain Enzyme Assays	Lactate, Pyruvate, Lactate:Pyruvate ratio, Creatine kinase, Creatine, and Amino acids	[93,94,95]
Mitochondrial DNA Analysis	miR-27b-3p	[96]
	POLG, DNM1L gene (c.1207C>T), MIPEP c.304C>T and m.10225T>G	[97,98]
	3243A>G/MT-TL1	[94]
	Circulating cell-free mitochondrial DNA (ccf mtDNA)	[99]
Omic Profiling	NDUFS3, COX2, CALR, SORT, COX6B1, COX6C, TXN2, SOD2, TFAM, SDHC, CS, MT-CO1, MT-CO2, TOMM70A, TOMM20, and RAB1A	[95,100,101]
Imaging Techniques		[94,102]

## Data Availability

No new data was created.

## References

[1] Iheagwam F.N., Iheagwam O.T., Onuoha M.K., Ogunlana O.O., Chinedu S.N. (2022). *Terminalia catappa* aqueous leaf extract reverses insulin resistance, improves glucose transport and activates PI3K/AKT signalling in high fat/streptozotocin-induced diabetic rats. Sci. Rep..

[2] Zajec A., Trebušak Podkrajšek K., Tesovnik T., Šket R., Čugalj Kern B., Jenko Bizjan B., Šmigoc Schweiger D., Battelino T., Kovač J. (2022). Pathogenesis of type 1 diabetes: Established facts and new insights. Genes.

[3] Iheagwam F.N., Odiba J.K., Iheagwam O.T., Ogunlana O.O., Chinedu S.N. (2021). Type 2 diabetes mellitus mediation by the disruptive activity of environmental toxicants on sex hormone receptors: In silico evaluation. Toxics.

[4] Iheagwam F.N., Iheagwam O.T., Ogunlana O.O., Chinedu S.N. (2023). *Terminalia catappa* leaf abrogates diabetes-induced dyslipidaemia in type 2 diabetic rats by upregulating lipid metabolic genes. Gene Expr..

[5] Sharma A.K., Singh S., Singh H., Mahajan D., Kolli P., Mandadapu G., Kumar B., Kumar D., Kumar S., Jena M.K. (2022). Deep insight of the pathophysiology of gestational diabetes mellitus. Cells.

[6] Amelia R., Harahap J., Fujiati I.I., Wijaya H., Zulham (2024). Educational model and prevention on prediabetes: A systematic review. Curr. Diabetes Rev..

[7] Zhang H., Colclough K., Gloyn A.L., Pollin T.I. (2021). Monogenic diabetes: A gateway to precision medicine in diabetes. J. Clin. Investig..

[8] Rivera Nieves A.M., Wauford B.M., Fu A. (2024). Mitochondrial bioenergetics, metabolism, and beyond in pancreatic β-cells and diabetes. Front. Mol. Biosci..

[9] Luo J.-S., Ning J.-Q., Chen Z.-Y., Li W.-J., Zhou R.-L., Yan R.-Y., Chen M.-J., Ding L.-L. (2022). The role of mitochondrial quality control in cognitive dysfunction in diabetes. Neurochem. Res..

[10] Ding W., Yang X., Lai K., Jiang Y., Liu Y. (2024). The potential of therapeutic strategies targeting mitochondrial biogenesis for the treatment of insulin resistance and type 2 diabetes mellitus. Arch. Pharm. Res..

[11] Yang L., Han W., Luo Y., Hu X., Xu Y., Li H., Hu C., Huang D., Ma J., Yang Y. (2018). Adapentpronitrile, a new dipeptidyl peptidase-IV inhibitor, ameliorates diabetic neuronal injury through inhibiting mitochondria-related oxidative stress and apoptosis. Front. Cell. Neurosci..

[12] Pearce B., Pearce K. (2024). Mitochondrial dysfunction and diabetes in South Africa: A review. Endocr. Metab. Sci..

[13] Zhang Z., Huang Q., Zhao D., Lian F., Li X., Qi W. (2023). The impact of oxidative stress-induced mitochondrial dysfunction on diabetic microvascular complications. Front. Endocrinol..

[14] Cojocaru K.-A., Luchian I., Goriuc A., Antoci L.-M., Ciobanu C.-G., Popescu R., Vlad C.-E., Blaj M., Foia L.G. (2023). Mitochondrial dysfunction, oxidative stress, and therapeutic strategies in diabetes, obesity, and cardiovascular disease. Antioxidants.

[15] Bhatti J.S., Sehrawat A., Mishra J., Sidhu I.S., Navik U., Khullar N., Kumar S., Bhatti G.K., Reddy P.H. (2022). Oxidative stress in the pathophysiology of type 2 diabetes and related complications: Current therapeutics strategies and future perspectives. Free Radic. Biol. Med..

[16] Caturano A., D’Angelo M., Mormone A., Russo V., Mollica M.P., Salvatore T., Galiero R., Rinaldi L., Vetrano E., Marfella R. (2023). Oxidative stress in type 2 diabetes: Impacts from pathogenesis to lifestyle modifications. Curr. Issues Mol. Biol..

[17] Antonucci S., Di Lisa F., Kaludercic N. (2021). Mitochondrial reactive oxygen species in physiology and disease. Cell Calcium.

[18] Bayliak M.M., Gospodaryov D.V., Lushchak V.I. (2022). Mimicking caloric restriction for anti-aging effects: The pro-oxidant role of alpha-ketoglutarate. Curr. Opin. Toxicol..

[19] Brand M.D. (2016). Mitochondrial generation of superoxide and hydrogen peroxide as the source of mitochondrial redox signaling. Free Radic. Biol. Med..

[20] Selim L.A., Hassaan H., El-Khamisy S. (2017). Mitochondrial Diseases as Model of Neurodegeneration. Personalised Medicine: Lessons from Neurodegeneration to Cancer.

[21] Lv B., Bao X., Li P., Lian J., Wu Y., An T., Zhang J., Yang X., Wang T., Zhu J. (2020). Transcriptome Sequencing Analysis of Peripheral Blood of Type 2 Diabetes Mellitus Patients with Thirst and Fatigue. Front. Endocrinol..

[22] Dabravolski S.A., Orekhova V.A., Baig M.S., Bezsonov E.E., Starodubova A.V., Popkova T.V., Orekhov A.N. (2021). The role of mitochondrial mutations and chronic inflammation in diabetes. Int. J. Mol. Sci..

[23] Al-Ghamdi B.A., Al-Shamrani J.M., El-Shehawi A.M., Al-Johani I., Al-Otaibi B.G. (2022). Role of mitochondrial DNA in diabetes mellitus type I and type II. Saudi J. Biol. Sci..

[24] Zeng X., Huang Q., Long S.L., Zhong Q., Mo Z. (2020). Mitochondrial Dysfunction in Polycystic Ovary Syndrome. DNA Cell Biol..

[25] Lima J.E.B.F., Moreira N.C.S., Sakamoto-Hojo E.T. (2022). Mechanisms underlying the pathophysiology of type 2 diabetes: From risk factors to oxidative stress, metabolic dysfunction, and hyperglycemia. Mutat. Res. Genet. Toxicol. Environ. Mutagen..

[26] Sangwung P., Petersen K.F., Shulman G.I., Knowles J.W. (2020). Mitochondrial Dysfunction, Insulin Resistance, and Potential Genetic Implications. Endocrinology.

[27] Jackson D.N., Theiss A.L. (2020). Gut bacteria signaling to mitochondria in intestinal inflammation and cancer. Gut Microbes.

[28] Sikalidis A.K., Maykish A. (2020). The gut microbiome and type 2 diabetes mellitus: Discussing a complex relationship. Biomedicines.

[29] Vezza T., Abad-Jiménez Z., Marti-Cabrera M., Rocha M., Víctor V.M. (2020). Microbiota-mitochondria inter-talk: A potential therapeutic strategy in obesity and type 2 diabetes. Antioxidants.

[30] Duan L., An X., Zhang Y., Jin D., Zhao S., Zhou R., Duan Y., Zhang Y., Liu X., Lian F. (2021). Gut microbiota as the critical correlation of polycystic ovary syndrome and type 2 diabetes mellitus. Biomed. Pharmacother..

[31] Anderson G. (2023). Type I diabetes pathoetiology and pathophysiology: Roles of the gut microbiome, pancreatic cellular interactions, and the ‘bystander’ activation of memory CD8+ T cells. Int. J. Mol. Sci..

[32] Zhao R.-Z., Jiang S., Zhang L., Yu Z.-B. (2019). Mitochondrial electron transport chain, ROS generation and uncoupling (Review). Int. J. Mol. Med..

[33] Monteiro B.S., Freire-Brito L., Carrageta D.F., Oliveira P.F., Alves M.G. (2021). Mitochondrial uncoupling proteins (UCPs) as key modulators of ros homeostasis: A crosstalk between diabesity and male infertility?. Antioxidants.

[34] Čater M., Bombek L.K. (2022). Protective role of mitochondrial uncoupling proteins against age-related oxidative stress in type 2 diabetes mellitus. Antioxidants.

[35] Vilas-Boas E.A., Almeida D.C., Roma L.P., Ortis F., Carpinelli A.R. (2021). Lipotoxicity and β-cell failure in type 2 diabetes: Oxidative stress linked to NADPH oxidase and ER stress. Cells.

[36] Galicia-Garcia U., Benito-Vicente A., Jebari S., Larrea-Sebal A., Siddiqi H., Uribe K.B., Ostolaza H., Martín C. (2020). Pathophysiology of type 2 diabetes mellitus. Int. J. Mol. Sci..

[37] Ding X., Fang T., Pang X., Pan X., Tong A., Lin Z., Zheng S., Zheng N. (2023). Mitochondrial DNA abnormalities and metabolic syndrome. Front. Cell Dev. Biol..

[38] Todosenko N., Khaziakhmatova O., Malashchenko V., Yurova K., Bograya M., Beletskaya M., Vulf M., Gazatova N., Litvinova L. (2023). Mitochondrial dysfunction associated with mtDNA in metabolic syndrome and obesity. Int. J. Mol. Sci..

[39] Sultana M.A., Hia R.A., Akinsiku O., Hegde V. (2023). Peripheral mitochondrial dysfunction: A potential contributor to the development of metabolic disorders and Alzheimer’s disease. Biology.

[40] McMeekin L.J., Fox S.N., Boas S.M., Cowell R.M. (2021). Dysregulation of PGC-1α-dependent transcriptional programs in neurological and developmental disorders: Therapeutic challenges and opportunities. Cells.

[41] Rius-Pérez S., Torres-Cuevas I., Millán I., Ortega Á.L., Pérez S. (2020). PGC-1α, inflammation, and oxidative stress: An integrative view in metabolism. Oxid. Med. Cell Longev..

[42] Chen W., Zhao H., Li Y. (2023). Mitochondrial dynamics in health and disease: Mechanisms and potential targets. Sig Transduct. Target. Ther..

[43] Vezza T., Díaz-Pozo P., Canet F., de Marañón A.M., Abad-Jiménez Z., García-Gargallo C., Roldan I., Solá E., Bañuls C., López-Domènech S. (2022). The role of mitochondrial dynamic dysfunction in age-associated type 2 diabetes. World J. Mens. Health.

[44] Yu T., Wang L., Zhang L., Deuster P.A. (2023). Mitochondrial fission as a therapeutic target for metabolic diseases: Insights into antioxidant strategies. Antioxidants.

[45] Iheagwam F.N., Batiha G.E.-S., Ogunlana O.O., Chinedu S.N. (2021). *Terminalia* catappa extract palliates redox imbalance and inflammation in diabetic rats by upregulating Nrf-2 gene. Int. J. Inflamm..

[46] Fex M., Nicholas L.M., Vishnu N., Medina A., Sharoyko V.V., Nicholls D.G., Spégel P., Mulder H. (2018). The pathogenetic role of β-cell mitochondria in type 2 diabetes. J. Endocrinol..

[47] Prasun P. (2020). Mitochondrial dysfunction in metabolic syndrome. Biochim. Biophys. Acta Mol. Basis Dis..

[48] Salvatore T., Pafundi P.C., Galiero R., Albanese G., Di Martino A., Caturano A., Vetrano E., Rinaldi L., Sasso F.C. (2021). The diabetic cardiomyopathy: The contributing pathophysiological mechanisms. Front. Med..

[49] Deng Y.-W., Liu F., Li Z.-T., Gao J.-H., Zhao Y., Yang X.-L., Xia Y.-L. (2022). Hyperglycemia promotes myocardial dysfunction via the ERS-MAPK10 signaling pathway in db/db mice. Lab. Investig..

[50] Masenga S.K., Kabwe L.S., Chakulya M., Kirabo A. (2023). Mechanisms of oxidative stress in metabolic syndrome. Int. J. Mol. Sci..

[51] Gollmer J., Zirlik A., Bugger H. (2020). Mitochondrial Mechanisms in Diabetic Cardiomyopathy. Diabetes Metab. J..

[52] Zamora M., Villena J.A. (2019). Contribution of Impaired Insulin Signaling to the Pathogenesis of Diabetic Cardiomyopathy. Int. J. Mol. Sci..

[53] Dia M., Gomez L., Thibault H., Tessier N., Leon C., Chouabe C., Ducreux S., Gallo-Bona N., Tubbs E., Bendridi N. (2020). Reduced reticulum–mitochondria Ca^2+^ transfer is an early and reversible trigger of mitochondrial dysfunctions in diabetic cardiomyopathy. Basic. Res. Cardiol..

[54] Sagoo M.K., Gnudi L. (2020). Diabetic nephropathy: An overview. Methods Mol. Biol..

[55] Agarwal R. (2021). Pathogenesis of diabetic nephropathy. ADA Clin. Compend..

[56] Ames M.K., Atkins C.E., Pitt B. (2019). The renin-angiotensin-aldosterone system and its suppression. J. Vet. Intern. Med..

[57] Zhao D., Zhong R., Wang X., Yan Z. (2024). Mitochondrial dysfunction in diabetic nephropathy: Insights and therapeutic avenues from traditional Chinese medicine. Front. Endocrinol..

[58] Yao L., Liang X., Qiao Y., Chen B., Wang P., Liu Z. (2022). Mitochondrial dysfunction in diabetic tubulopathy. Metabolism.

[59] Tomita I., Kume S., Sugahara S., Osawa N., Yamahara K., Yasuda-Yamahara M., Takeda N., Chin-Kanasaki M., Kaneko T., Mayoux E. (2020). SGLT2 Inhibition Mediates Protection from Diabetic Kidney Disease by Promoting Ketone Body-Induced mTORC1 Inhibition. Cell Metab..

[60] Liu S., Han S., Wang C., Chen H., Xu Q., Feng S., Wang Y., Yao J., Zhou Q., Tang X. (2024). MAPK1 Mediates MAM Disruption and Mitochondrial Dysfunction in Diabetic Kidney Disease via the PACS-2-Dependent Mechanism. Int. J. Biol. Sci..

[61] Darenskaya M., Kolesnikov S., Semenova N., Kolesnikova L. (2023). Diabetic nephropathy: Significance of determining oxidative stress and opportunities for antioxidant therapies. Int. J. Mol. Sci..

[62] Amorim R.G., Guedes G.D.S., Vasconcelos S.M.L., Santos J.C.D.F. (2019). Kidney disease in diabetes mellitus: Cross-linking between hyperglycemia, redox imbalance and inflammation. Arq. Bras. Cardiol..

[63] Strand N., Anderson M.A., Attanti S., Gill B., Wie C., Dawodu A., Pagan-Rosado R., Harbell M.W., Maloney J.A. (2024). Diabetic Neuropathy: Pathophysiology Review. Curr. Pain. Headache Rep..

[64] Feldman E.L., Callaghan B.C., Pop-Busui R., Zochodne D.W., Wright D.E., Bennett D.L., Bril V., Russell J.W., Viswanathan V. (2019). Diabetic neuropathy. Nat. Rev. Dis. Primers.

[65] Khan I., Preeti K., Kumar R., Kumar Khatri D., Bala Singh S. (2023). Piceatannol promotes neuroprotection by inducing mitophagy and mitobiogenesis in the experimental diabetic peripheral neuropathy and hyperglycemia-induced neurotoxicity. Int. Immunopharmacol..

[66] Yuan P., Song F., Zhu P., Fan K., Liao Q., Huang L., Liu Z. (2022). Poly (ADP-ribose) polymerase 1-mediated defective mitophagy contributes to painful diabetic neuropathy in the db/db model. J. Neurochem..

[67] He J., Qin Z., Chen X., He W., Li D., Zhang L., Le Y., Xiong Q., Zhang B., Wang H. (2022). HIF-1α Ameliorates Diabetic Neuropathic Pain via Parkin-Mediated Mitophagy in a Mouse Model. Biomed. Res. Int..

[68] Yang C., Zhao X., An X., Zhang Y., Sun W., Zhang Y., Duan Y., Kang X., Sun Y., Jiang L. (2023). Axonal transport deficits in the pathogenesis of diabetic peripheral neuropathy. Front. Endocrinol..

[69] Yeoh S., Warner W.S., Merchant S.S., Hsu E.W., Agoston D.v., Mahan M.A. (2022). Incorporating blood flow in nerve injury and regeneration assessment. Front. Surg..

[70] Hushmandi K., Einollahi B., Aow R., Suhairi S.B., Klionsky D.J., Aref A.R., Reiter R.J., Makvandi P., Rabiee N., Xu Y. (2024). Investigating the Interplay between Mitophagy and Diabetic Neuropathy: Uncovering the hidden secrets of the disease pathology. Pharmacol. Res..

[71] Kowluru R.A. (2020). Diabetic Retinopathy: Mitochondria Caught in a Muddle of Homocysteine. J. Clin. Med..

[72] Wu Y., Zou H. (2022). Research Progress on Mitochondrial Dysfunction in Diabetic Retinopathy. Antioxidants.

[73] Kowluru R.A., Shan Y., Mishra M. (2016). Dynamic DNA methylation of matrix metalloproteinase-9 in the development of diabetic retinopathy. Lab. Investig..

[74] Mohammad G., Kowluru R.A. (2020). Homocysteine Disrupts Balance between MMP-9 and Its Tissue Inhibitor in Diabetic Retinopathy: The Role of DNA Methylation. Int. J. Mol. Sci..

[75] Kowluru R.A. (2019). Mitochondrial Stability in Diabetic Retinopathy: Lessons Learned from Epigenetics. Diabetes.

[76] Shu D.Y., Chaudhary S., Cho K.-S., Lennikov A., Miller W.P., Thorn D.C., Yang M., McKay T.B. (2023). Role of oxidative stress in ocular diseases: A balancing act. Metabolites.

[77] Fu M., Peng D., Lan T., Wei Y., Wei X. (2022). Multifunctional regulatory protein connective tissue growth factor (CTGF): A potential therapeutic target for diverse diseases. Acta Pharm. Sin. B.

[78] Turton N., Cufflin N., Dewsbury M., Fitzpatrick O., Islam R., Watler L.L., McPartland C., Whitelaw S., Connor C., Morris C. (2022). The biochemical assessment of mitochondrial respiratory chain disorders. Int. J. Mol. Sci..

[79] Avram V.F., Merce A.P., Hâncu I.M., Bătrân A.D., Kennedy G., Rosca M.G., Muntean D.M. (2022). Impairment of mitochondrial respiration in metabolic diseases: An overview. Int. J. Mol. Sci..

[80] Kaludercic N., Di Lisa F. (2020). Mitochondrial ROS formation in the pathogenesis of diabetic cardiomyopathy. Front. Cardiovasc. Med..

[81] Frazier A.E., Vincent A.E., Turnbull D.M., Thorburn D.R., Taylor R.W. (2020). Assessment of mitochondrial respiratory chain enzymes in cells and tissues. Methods Cell Biol..

[82] Qiu S., Cai Y., Yao H., Lin C., Xie Y., Tang S., Zhang A. (2023). Small molecule metabolites: Discovery of biomarkers and therapeutic targets. Signal Transduct. Target. Ther..

[83] Jin Y., Ji W., Yang H., Chen S., Zhang W., Duan G. (2020). Endothelial activation and dysfunction in COVID-19: From basic mechanisms to potential therapeutic approaches. Sig Transduct. Target. Ther..

[84] Tanase D.M., Gosav E.M., Botoc T., Floria M., Tarniceriu C.C., Maranduca M.A., Haisan A., Cucu A.I., Rezus C., Costea C.F. (2023). Depiction of branched-chain amino acids (BCAAS) in diabetes with a focus on diabetic microvascular complications. J. Clin. Med..

[85] Hussain S., Mubeen I., Ullah N., Shah S.S.U.D., Khan B.A., Zahoor M., Ullah R., Khan F.A., Sultan M.A. (2022). Modern diagnostic imaging technique applications and risk factors in the medical field: A review. Biomed. Res. Int..

[86] Tersalvi G., Beltrani V., Grübler M.R., Molteni A., Cristoforetti Y., Pedrazzini G., Treglia G., Biasco L. (2023). Positron emission tomography in heart failure: From pathophysiology to clinical application. J. Cardiovasc. Dev. Dis..

[87] Wu M., Junker D., Branca R.T., Karampinos D.C. (2020). Magnetic resonance imaging techniques for brown adipose tissue detection. Front. Endocrinol..

[88] Donati A., Damiani E., Domizi R., Scorcella C., Carsetti A., Tondi S., Monaldi V., Adrario E., Romano R., Pelaia P. (2016). Near-infrared spectroscopy for assessing tissue oxygenation and microvascular reactivity in critically ill patients: A prospective observational study. Crit. Care.

[89] Chen Z.-Z., Gerszten R.E. (2020). Metabolomics and proteomics in type 2 diabetes. Circ. Res..

[90] FDA-NIH Biomarker Working Group (2016). BEST (Biomarkers, EndpointS, and other Tools) Resource.

[91] Lan L.Y.-L., Kumar W.M., Liu L.S., Roberts A.K., Chen S., Snyder M. (2024). Biomarkers in precision medicine. Biosensors in Precision Medicine.

[92] Matthews D.R., Li Q., Perkovic V., Mahaffey K.W., de Zeeuw D., Fulcher G., Desai M., Hiatt W.R., Nehler M., Fabbrini E. (2019). Effects of canagliflozin on amputation risk in type 2 diabetes: The CANVAS Program. Diabetologia.

[93] Varhaug K.N., Hikmat O., Nakkestad H.L., Vedeler C.A., Bindoff L.A. (2021). Serum biomarkers in primary mitochondrial disorders. Brain Commun..

[94] Evangelisti S., Gramegna L.L., La Morgia C., Di Vito L., Maresca A., Talozzi L., Bianchini C., Mitolo M., Manners D.N., Caporali L. (2022). Molecular biomarkers correlate with brain grey and white matter changes in patients with mitochondrial m.3243A>G mutation. Mol. Genet. Metab..

[95] Shayota B.J. (2024). Biomarkers of mitochondrial disorders. Neurotherapeutics.

[96] Wang W., Zhuang Q., Ji K., Wen B., Lin P., Zhao Y., Li W., Yan C. (2017). Identification of miRNA, lncRNA and mRNA-associated ceRNA networks and potential biomarker for MELAS with mitochondrial DNA A3243G mutation. Sci. Rep..

[97] Wu T., He F., Xiao N., Han Y., Yang L., Peng J. (2022). Phenotype-Genotype Analysis Based on Molecular Classification in 135 Children with Mitochondrial Disease. Pediatr. Neurol..

[98] Peñas A., Fernández-De la Torre M., Laine-Menéndez S., Lora D., Illescas M., García-Bartolomé A., Morales-Conejo M., Arenas J., Martín M.A., Morán M. (2021). Plasma Gelsolin Reinforces the Diagnostic Value of FGF-21 and GDF-15 for Mitochondrial Disorders. Int. J. Mol. Sci..

[99] Trifunov S., Paredes-Fuentes A.J., Badosa C., Codina A., Montoya J., Ruiz-Pesini E., Jou C., Garrabou G., Grau-Junyent J.M., Yubero D. (2021). Circulating Cell-Free Mitochondrial DNA in Cerebrospinal Fluid as a Biomarker for Mitochondrial Diseases. Clin. Chem..

[100] Chae S., Kim S.-J., Do Koo Y., Lee J.H., Kim H., Ahn B.Y., Ha Y.-C., Kim Y.-H., Jang M.G., Koo K.-H. (2018). A mitochondrial proteome profile indicative of type 2 diabetes mellitus in skeletal muscles. Exp. Mol. Med..

[101] Gómez-Serrano M., Camafeita E., López J.A., Rubio M.A., Bretón I., García-Consuegra I., García-Santos E., Lago J., Sánchez-Pernaute A., Torres A. (2017). Differential proteomic and oxidative profiles unveil dysfunctional protein import to adipocyte mitochondria in obesity-associated aging and diabetes. Redox Biol..

[102] Lunsing R.J., Strating K., de Koning T.J., Sijens P.E. (2017). Diagnostic value of MRS-quantified brain tissue lactate level in identifying children with mitochondrial disorders. Eur. Radiol..

[103] Jiang Q., Yin J., Chen J., Ma X., Wu M., Liu G., Yao K., Tan B., Yin Y. (2020). Mitochondria-targeted antioxidants: A step towards disease treatment. Oxid. Med. Cell Longev..

[104] Napolitano G., Fasciolo G., Venditti P. (2021). Mitochondrial management of reactive oxygen species. Antioxidants.

[105] Sulaimon L.A., Afolabi L.O., Adisa R.A., Ayankojo A.G., Afolabi M.O., Adewolu A.M., Wan X. (2022). Pharmacological significance of MitoQ in ameliorating mitochondria-related diseases. Adv. Redox Biol..

[106] Mason S.A., Wadley G.D., Keske M.A., Parker L. (2022). Effect of mitochondrial-targeted antioxidants on glycaemic control, cardiovascular health, and oxidative stress in humans: A systematic review and meta-analysis of randomized controlled trials. Diabetes Obes. Metab..

[107] Krako Jakovljevic N., Pavlovic K., Jotic A., Lalic K., Stoiljkovic M., Lukic L., Milicic T., Macesic M., Stanarcic Gajovic J., Lalic N.M. (2021). Targeting Mitochondria in Diabetes. Int. J. Mol. Sci..

[108] Most J., Timmers S., Warnke I., Jocken J.W., van Boekschoten M., de Groot P., Bendik I., Schrauwen P., Goossens G.H., Blaak E.E. (2016). Combined epigallocatechin-3-gallate and resveratrol supplementation for 12 wk increases mitochondrial capacity and fat oxidation, but not insulin sensitivity, in obese humans: A randomized controlled trial12. Am. J. Clin. Nutr..

[109] Pant T., Uche N., Juric M., Bosnjak Z.J. (2023). Clinical Relevance of lncRNA and Mitochondrial Targeted Antioxidants as Therapeutic Options in Regulating Oxidative Stress and Mitochondrial Function in Vascular Complications of Diabetes. Antioxidants.

[110] Ni R., Cao T., Xiong S., Ma J., Fan G.-C., Lacefield J.C., Lu Y., Le Tissier S., Peng T. (2016). Therapeutic inhibition of mitochondrial reactive oxygen species with mito-TEMPO reduces diabetic cardiomyopathy. Free Radic. Biol. Med..

[111] Xiong X., Lu W., Qin X., Luo Q., Zhou W. (2020). Downregulation of the GLP-1/CREB/adiponectin pathway is partially responsible for diabetes-induced dysregulated vascular tone and VSMC dysfunction. Biomed. Pharmacother..

[112] Ngo D.T.M., Sverdlov A.L., Karki S., Macartney-Coxson D., Stubbs R.S., Farb M.G., Carmine B., Hess D.T., Colucci W.S., Gokce N. (2019). Oxidative modifications of mitochondrial complex II are associated with insulin resistance of visceral fat in obesity. Am. J. Physiol. Endocrinol. Metab..

[113] Wu M., Liao L., Jiang L., Zhang C., Gao H., Qiao L., Liu S., Shi D. (2019). Liver-targeted Nano-MitoPBN normalizes glucose metabolism by improving mitochondrial redox balance. Biomaterials.

[114] Zhao X., An X., Yang C., Sun W., Ji H., Lian F. (2023). The crucial role and mechanism of insulin resistance in metabolic disease. Front. Endocrinol..

[115] de Marañón A.M., Díaz-Pozo P., Canet F., Díaz-Morales N., Abad-Jiménez Z., López-Domènech S., Vezza T., Apostolova N., Morillas C., Rocha M. (2022). Metformin modulates mitochondrial function and mitophagy in peripheral blood mononuclear cells from type 2 diabetic patients. Redox Biol..

[116] Fatima A., Rasool S., Devi S., Talha M., Waqar F., Nasir M., Khan M.R., Ibne Ali Jaffari S.M., Haider A., Shah S.U. (2023). Exploring the cardiovascular benefits of sodium-glucose cotransporter-2 (sglt2) inhibitors: Expanding horizons beyond diabetes management. Cureus.

[117] Johansson K.S., Brønden A., Knop F.K., Christensen M.B. (2020). Clinical pharmacology of imeglimin for the treatment of type 2 diabetes. Expert. Opin. Pharmacother..

[118] Crabtree T.S., DeFronzo R.A., Ryder R.E.J., Bailey C.J. (2020). Imeglimin, a novel, first in-class, blood glucose-lowering agent: A systematic review and meta-analysis of clinical evidence. Br. J. Diabetes.

[119] Vial G., Lamarche F., Cottet-Rousselle C., Hallakou-Bozec S., Borel A.-L., Fontaine E. (2021). The mechanism by which imeglimin inhibits gluconeogenesis in rat liver cells. Endocrinol. Diabetes Metab..

[120] El-Hattab A.W., Zarante A.M., Almannai M., Scaglia F. (2017). Therapies for mitochondrial diseases and current clinical trials. Mol. Genet. Metab..

[121] Russell O.M., Gorman G.S., Lightowlers R.N., Turnbull D.M. (2020). Mitochondrial diseases: Hope for the future. Cell.

[122] Di Donfrancesco A., Massaro G., Di Meo I., Tiranti V., Bottani E., Brunetti D. (2022). Gene therapy for mitochondrial diseases: Current status and future perspective. Pharmaceutics.

[123] Aravintha Siva M., Mahalakshmi R., Bhakta-Guha D., Guha G. (2019). Gene therapy for the mitochondrial genome: Purging mutations, pacifying ailments. Mitochondrion.

[124] Soldatov V.O., Kubekina M.V., Skorkina M.Y., Belykh A.E., Egorova T.V., Korokin M.V., Pokrovskiy M.V., Deykin A.V., Angelova P.R. (2022). Current advances in gene therapy of mitochondrial diseases. J. Transl. Med..

[125] Kar B., Castillo S.R., Sabharwal A., Clark K.J., Ekker S.C. (2023). Mitochondrial base editing: Recent advances towards therapeutic opportunities. Int. J. Mol. Sci..

[126] Coutinho E., Batista C., Sousa F., Queiroz J., Costa D. (2017). Mitochondrial Gene Therapy: Advances in Mitochondrial Gene Cloning, Plasmid Production, and Nanosystems Targeted to Mitochondria. Mol. Pharm..

[127] Faria R., Vivés E., Boisguerin P., Sousa A., Costa D. (2021). Development of Peptide-Based Nanoparticles for Mitochondrial Plasmid DNA Delivery. Polymers.

[128] Hirano M., Emmanuele V., Quinzii C.M. (2018). Emerging therapies for mitochondrial diseases. Essays Biochem..

[129] Faria R., Paul M., Biswas S., Vivès E., Boisguérin P., Sousa Â., Costa D. (2022). Peptides vs. Polymers: Searching for the Most Efficient Delivery System for Mitochondrial Gene Therapy. Pharmaceutics.

[130] Neves A.R., Albuquerque T., Faria R., Paul M., Biswas S., Sousa Â., Costa D. (2021). Development of Tailor-Made Dendrimer Ternary Complexes for Drug/Gene Co-Delivery in Cancer. Pharmaceutics.

[131] Zhu H., Bi D., Zhang Y., Kong C., Du J., Wu X., Wei Q., Qin H. (2022). Ketogenic diet for human diseases: The underlying mechanisms and potential for clinical implementations. Signal Transduct. Target. Ther..

[132] Al-Rawaf H.A., Gabr S.A., Iqbal A., Alghadir A.H. (2023). High-intensity interval training improves glycemic control, cellular apoptosis, and oxidative stress of type 2 diabetic patients. Medicina.

[133] Golubitzky A., Dan P., Weissman S., Link G., Wikstrom J.D., Saada A. (2011). Screening for Active Small Molecules in Mitochondrial Complex I Deficient Patient’s Fibroblasts, Reveals AICAR as the Most Beneficial Compound. PLoS ONE.

[134] Dai X., Shen L. (2022). Advances and trends in omics technology development. Front. Med..

[135] Auerbach B.J., Hu J., Reilly M.P., Li M. (2021). Applications of single-cell genomics and computational strategies to study common disease and population-level variation. Genome Res..

[136] Chen C., Wang J., Pan D., Wang X., Xu Y., Yan J., Wang L., Yang X., Yang M., Liu G. (2023). Applications of multi-omics analysis in human diseases. MedComm.

[137] Mittal R., Camick N., Lemos J.R.N., Hirani K. (2024). Gene-environment interaction in the pathophysiology of type 1 diabetes. Front. Endocrinol..

[138] Cefalu W.T., Andersen D.K., Arreaza-Rubín G., Pin C.L., Sato S., Verchere C.B., Woo M., Rosenblum N.D. (2022). Heterogeneity of diabetes: β-cells,phenotypes, and precision medicine: Proceedings of an International Symposium of the Canadian Institutes of Health Research’s Institute of Nutrition, Metabolism and Diabetes and the U.S. National Institutes of Health’s National Institute of Diabetes and Digestive and Kidney Diseases. Diabetes Care.

